# Recent advances in plant kinetochore research

**DOI:** 10.3389/fcell.2024.1510019

**Published:** 2025-01-22

**Authors:** Elena Kozgunova

**Affiliations:** ^1^ Institute for Advanced Research, Nagoya University, Nagoya, Japan; ^2^ Department of Biological Science, Graduate School of Science, Nagoya University, Nagoya, Japan

**Keywords:** kinetochore, cell division, spindle, chromosome, plant, mitosis

## Abstract

Faithful chromosome segregation is crucial for cell division in eukaryotes, facilitated by the kinetochore, a multi-subunit protein complex that connects chromosomes to the spindle microtubules. Recent research has significantly advanced our understanding of kinetochore function in plants, including surprising findings about spindle assembly checkpoint, the composition of the inner kinetochore and unique kinetochore arrangement in holocentric *Cuscuta* species. Additionally, some kinetochore proteins in plants have been implicated in roles beyond chromosome segregation, such as cytokinesis regulation and involvement in developmental processes. This review summarizes recent insights into plant kinetochore biology, compares plant kinetochores with those of animals and fungi, and highlights key open questions and potential future directions in the field.

## 1 Introduction

The kinetochore is a macromolecular protein complex that acts as an adapter between chromosomes and spindle microtubules. The kinetochore is typically divided into two regions: the inner kinetochore, which binds to the centromeric DNA, and the outer kinetochore, which interacts with microtubules (Musacchio and Desai, 2017; Ariyoshi and Fukagawa, 2023). Proper kinetochore attachment to the microtubules is monitored by the Spindle Assembly Checkpoint (SAC), which delays anaphase by inhibiting the anaphase-promoting complex/cyclosome (APC/C) until proper attachment and alignment are achieved, thereby ensuring accurate chromosome distribution to daughter cells (Komaki and Schnittger, 2016; McAinsh and Kops, 2023).

Centromere identity is typically defined by the presence of an epigenetic marker: a centromere-specific variant of histone 3, known as CENH3 or CENP-A (CENPA). Research on plant centromeres has gained significant traction in recent years, due to its practical relevance for plant breeding, including haploid induction via centromere-mediated genome elimination (Marimuthu et al., 2021; Quiroz et al., 2024) and the design of stably inherited synthetic chromosomes (Puchta and Houben, 2024; Wang et al., 2024). Several reviews highlighting advances in plant centromere research have been recently published (Chandra et al., 2024; Naish and Henderson, 2024); therefore we do not focus on centromeres or centromeric histone in this manuscript. Here, we summarize recent discoveries in plant kinetochore research across various species, compare them with animal and fungal kinetochores, and discuss unanswered questions in the field.

### 1.1 Unusual composition of the inner kinetochore in plants

The inner kinetochore, also known as the Constitutive Centromere-Associated Network (CCAN), comprises 16 subunits in human cells. Recent studies in yeast and human cells have shown that CENP-LN serves as the primary DNA-binding module, with other CCAN subunits enhancing DNA binding. CENP-C acts as a critical linker, connecting CCAN to CENP-A and the centromere (Musacchio and Desai, 2017; Yan et al., 2019; Pesenti et al., 2022). In some species, the number of CCAN proteins is highly reduced; for example, the inner kinetochores of *C. elegans* and *D. melanogaster* consist only of CENP-A and CENP-C, with the latter directly linking the centromere to the outer kinetochore (Drinnenberg et al., 2016).

In plants, homologues of the CCAN subunits CENP-C, CENP-O, CENP-S (MHF1), CENP-X (MHF2), and CENP-U (BIN4) have been identified (Table 1). In Arabidopsis CENP-C was shown to co-localize with the 180 bp centromeric regions of chromosomes throughout the cell cycle (Ogura et al., 2004). Another study combining biochemistry and *in vivo* analysis demonstrated that CENP-C binds DNA through a specific 122-amino-acid region in maize, with binding affinity enhanced in the presence of small single-stranded centromeric RNAs (Du et al., 2010), suggesting centromeric ssRNA may have a potential role in augmenting kinetochore formation. Another study has also demonstrated that the γ-tubulin complex protein 3-interacting proteins (GIPs), GIP1 and GIP2, are involved into the recruitment of both CENH3 and CENP-C to the centromeres (Batzenschlager et al., 2015).

**TABLE 1:** Kinetochore and kinetochore-related proteins in plants.

Gene alias used in this review	Alias decoding	Other common aliases	Description/function	Accession number (*A. thaliana*)	Accession number (*P. patens*)	References (only plants)
CENH3	centromeric histone H3	CENP-A	**centromere/nucleosome**	AT1G01370	Pp3c1_20640	Chandra et al. (2024) and Naish and Henderson (2024)
CENP-C	centromere protein C		**inner kinetochore, CCAN**	AT1G15660	Pp3c2_32580	Ogura et al. (2004), Nagaki et al. (2009), and Du et al. (2010)
CENP-O	centromere protein O		*inner kinetochore, CCAN ?*	AT5G10710	Pp3c5_16590; Pp3c6_9310	Kozgunova et al. (2019)
CENP-S	centromere protein S	MHF1	**FANCM pathway,** *inner kinetochore, CCAN ?*	AT5G50930	Pp3c2_1780	Singh et al. (2010), Dangel et al. (2014), Girard et al. (2014), Kozgunova et al. (2019), and Li et al. (2023)
CENP-X	centromere protein X	MHF2		AT1G78790	Pp3c15_7370	
CENP-U	centromere protein U	BIN4, MID, MIDGET	**topoisomerase VI complex,** *inner kinetochore, CCAN ?*	AT5G24630	Pp3c11_21910; Pp3c7_3940	Breuer et al. (2007) and Kirik et al. (2007)
KNL1	kinetochore-null protein 1		**KNL complex, central/outer kinetochore**	AT2G04235	Pp3c6_1750	Su et al. (2021), Deng et al. (2024a)
ZWINT	ZW10 interacting kinetochore protein		KNL complex, central/outer kinetochore	AT3G23910	Pp3c12_25780; Pp3c4_4010	Neumann et al. (2023)
Mis12	minichromosome instability 12		**Mis12 complex, central/outer kinetochore**	AT5G35520	Pp3c2_13760	Sato et al. (2005) and Nagaki et al. (2009)
Nnf1	Necessary for Nuclear Function 1	PMF1		AT4G19350	Pp3c23_7640	Allipra et al. (2022)
Nsl1	Nnf1 Synthetic Lethal					
Dsn1	Dosage Suppressor of Nnf1			AT3G27520	Pp3c3_35410; Pp3c8_2210	
KAK1	Kinetochore-Associated Kinesin 1	kinesin 7, CENP-E	**Outer kinetochore, microtubule biding**	At1g59540	Pp3c6_21870	Hoopen et al. (2002), Miki et al. (2014), and Tang et al. (2024)
Ndc80	nuclear division cycle 80	HEC1	**Ndc80 complex, outer kinetochore, microtubule binding**	AT3G54630	Pp3c7_8870; Pp3c11_11580	Du and Dawe (2007) and Li et al. (2021)
Nuf2	nuclear filament-containing protein-2			AT1G61000	Pp3c12_6220	Li et al. (2021)
Spc24	spindle pole component 24	MUN1		AT3G08880; AT5G01570	Pp3c4_17930	Shin et al. (2018)
Spc25	spindle pole component 25			AT3G48210	Pp3c8_1270; Pp3c3_37370	Li et al. (2021)
SKA1	spindle and kinetochore–associated 1		**SKA complex. outer kinetochore, microtubule binding**	AT3G60660	Pp3c6_12030	Kozgunova et al. (2019)
SKA2	spindle and kinetochore–associated 2			AT2G24970	Pp3c17_11010; Pp3c14_9080	
SKA3	spindle and kinetochore–associated 3			AT5G06590	Pp3c26_10880; Pp3c4_24350	
Aurora3	Aurora kinase 3	AUR3	**Chromosome Passenger Complex (CPC)**	AT2G45490		Demidov et al. (2005)
BORR	BOREALIN RELATED	Borealin		AT3G02400	Pp3c20_6090	Komaki et al. (2020)
INCENP	Inner centromere protein			AT5G55820	Pp3c1_42830	
BORI1	BOREALIN RELATED INTERACTOR 1	FHA3		AT3G02400	Pp3c19_11080	Komaki et al. (2022)
BORI2	BOREALIN RELATED INTERACTOR 2			AT4G14490	Pp3c18_4140	
Mps1	MonoPolar Spindle		**Spindle Assembly Checkpoint (SAC)**	AT1G77720	Pp3c6_320; Pp3c16_20900	Lermontova et al. (2008), Caillaud et al. (2009), Ding et al. (2012), and Komaki and Schnittger (2017)
MAD1	Mitotic Arrest-Deficient 1	NES1		AT5G49880	Pp3c17_20400	
MAD2	Mitotic Arrest-Deficient 2			AT3G25980	Pp3c4_13910	
BMF1	BUB1/MAD3 FAMILY 1	BUB1		AT2G20635	Pp3c20_12130	
BMF2	BUB1/MAD3 FAMILY 2	BUBR1/MAD3		AT2G33560	Pp3c24_5040	
BMF3	BUB1/MAD3 FAMILY 3	BUBR1/MAD3		At5g05510		
BUB3; 3	Budding Uninhibited by Benzimidazole			At1g69400		Deng et al. (2024b)
BUB3; 1	Budding Uninhibited by Benzimidazole		**Phragmoplast microtubule dynamics**, *spindle assembly checkpoint*	At3g19590		Zhang et al. (2018)
BUB3; 2	Budding Uninhibited by Benzimidazole			At1g49910		

Bold text is used when protein function is supported by experimental evidence, italics is used when function based on homology contradicts or is not supported by experimental evidence. Accession numbers refer to TAIR, database for *A. thaliana* and Phytozome for *P. patens*.

Information about whether other CCAN proteins beside CENP-C play a role in plants’ kinetochore formation and cell division remains limited. CENP-U homologue is known as BIN4 or MIDGET, a part of the topoisomerase VI complex in plants. Mutations in BIN4 affect endoreduplication and produce brassinosteroid-insensitive dwarves with no known implications for kinetochore assembly or cell division (Breuer et al., 2007; Kirik et al., 2007). In human cells, CENP-U, as a part of CENP-OPQUR complex, recruits Polo-like kinase 1 (PLK1) to the kinetochore (Chen et al., 2021; Nguyen et al., 2021; Singh et al., 2021); the divergent role of the plant CENP-U is consistent with the absence of PLK1 in plant genomes.

CENP-S (MHF1) and CENP-X (MHF2) have been identified through genetic screening in Arabidopsis as factors that limit crossovers during meiosis (Girard et al., 2014), with similar results later shown in rice (Li et al., 2023). Another study has shown that MHF1 also works in the interstrand cross-link repair and is necessary for efficient homologous recombination (HR) in somatic cells (Dangel et al., 2014). A screen of kinetochore proteins in the bryophyte *Physcomitrium patens* (Physcomitrella) discovered that in moss cells, CENP-O, CENP-S, and CENP-X do not localize to kinetochores. Surprisingly, despite CENP-X’s lack of kinetochore localization, its knockdown via inducible RNA interference leads to chromosome missegregation defects during mitosis, resembling the phenotypes seen after knockdown of other kinetochore proteins (Kozgunova et al., 2019). Overall, the function of CENP-S and CENP-X in DNA repair and restricting meiotic crossovers appears to be highly conserved among eukaryotes (Singh et al., 2010), while their role in the inner kinetochore in plant cells remains ambiguous and calls for further investigation.

### 1.2 Outer kinetochore proteins in plants

**FIGURE 1:**
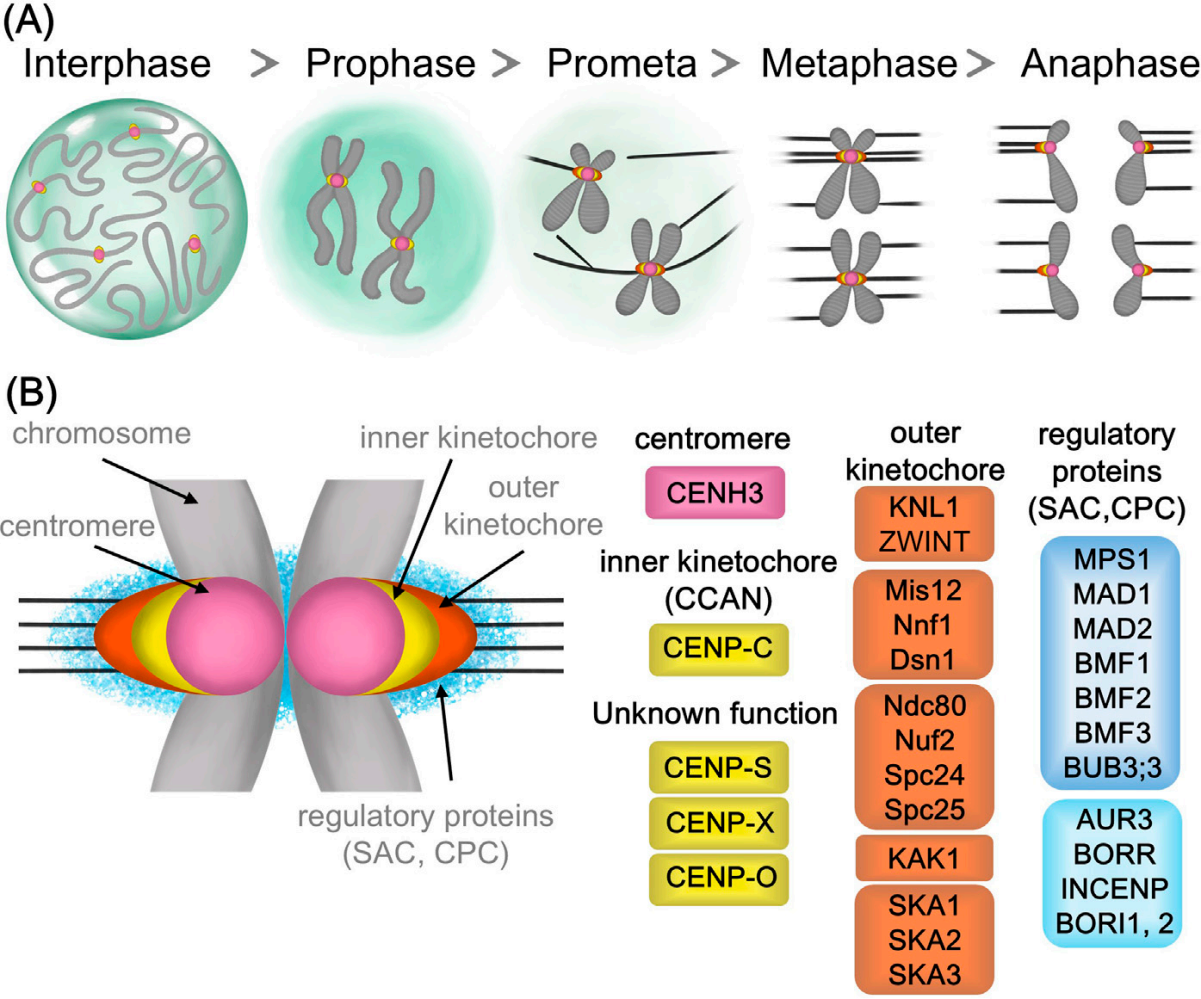
Cell division and kinetochore proteins in plant cells **(A)** Schematic of cell division stages and chromosome attachment to spindle microtubules by kinetochore **(B)** A color-coded model of kinetochore organization, showing centromere, inner kinetochore, outer kinetochore and regulatory proteins.

The outer kinetochore connects the inner kinetochore to microtubules, transmitting forces from microtubule depolymerization to move chromosomes. Known as the KMN protein assembly, it includes the KNL1, Mis12, and Ndc80 complexes, along with other important proteins like the SKA complex. Unlike the reduced inner kinetochore, outer kinetochore components are mostly conserved in plants (Figure 1; Table 1).

The Mis12 complex (MIND complex in *S. cerevisiae*), comprised of Mis12, Dsn1, Nuf2, and Nsl1 proteins, connects the inner and outer kinetochores by binding to CENP-C through Mis12 subunit, and the Ndc80 complex through Nsl1 and Dsn1 subunits, respectively (Musacchio and Desai, 2017). In Arabidopsis, a homologue of Mis12 co-localize with centromeric repeats throughout the cell cycle (Sato et al., 2005). Knockout of Nnf1 leads to embryonic lethality, indicating that it is essential for cell division. Interestingly, ectopic expression of GFP-Nnf1 rescues this lethality in homozygous *Nnf1−/−* mutants but results in dwarf plants, which is associated with decreased levels of endogenous polyamines (Allipra et al., 2022). The same study also suggested that the leucine zipper-like binding motif of Nnf1 may play a role in gibberellic acid (GA) metabolism. These findings highlight the intriguing possibility that kinetochore proteins can have additional functions beyond their roles in chromosome segregation.

The KNL1-ZWINT complex is known to recruits mitotic regulators, including SAC proteins BUBR1 and BUB1, to ensure accurate chromosome segregation. KNL1 features MELT-like motifs that, when phosphorylated, serve as docking sites for BUB1. However, in land plants, KNL1 lacks MELT repeats (Tromer et al., 2015), and the BUB1/MAD3 family has independently evolved distinct structures compared to animals and yeast (Komaki and Schnittger, 2016). This raises questions about how SAC proteins are recruited to the kinetochore and their binding partners. A recent study on maize identified KNL1 as a constitutive kinetochore component with an important role in chromosome segregation. A 145-amino acid region within maize KNL1 was found to interact with SAC proteins BMF1 and BMF2, but not BMF3 (Su et al., 2021). Interestingly, this BMF-interacting region is conserved in monocots but varies significantly in eudicots, suggesting different modes of SAC-to-kinetochore connections across plant lineages. This hypothesis was further confirmed in a study characterizing KNL1 homologue in Arabidopsis. The *knl1* null mutant is viable but shows severe growth defects and chromosome missegregation phenotype. In the mutant, the kinetochore localization of several SAC components, including BUB3.3, BMF3, and MAD1, is lost, while BMF1 remains unaffected. The authors also identified a eudicot-specific domain responsible for recruiting BUB3.3 and BMF3 to the kinetochore. Cross-species rescue experiments confirmed that KNL1 from the dicot plant (tomato) could recover the phenotype in the Arabidopsis *knl1* mutant, but KNL1 from monocots (rice) or bryophytes (*P*. *patens*) could not (Deng et al., 2024a). In animal cells, ZWINT recruits the RZZ complex, which facilitates the localization of dynein and dynactin to kinetochores. Neither dynein, dynactin, nor RZZ complex have been identified in plants. However, potential ZWINT homologues have been identified in *Cuscuta* and *Arabidopsis*, though their functions remain uncharacterized (Neumann et al., 2023).

Accurate chromosome segregation depends on stable end-on attachments between kinetochores and microtubules, with the microtubules’ plus-ends embedded in the kinetochore. The Ndc80 complex, a heterotetramer of Ndc80 (HEC1), Nuf2, Spc24, and Spc25, plays a crucial role in forming these attachments. The N-terminal regions of Ndc80 and Nuf2 mediate microtubule binding, while Spc24 and Spc25 connect to the Mis12 complex (Musacchio and Desai, 2017). In Arabidopsis, null mutants of Spc24 (meristem unstructured-1, MUN1) and Nuf2 are embryonically lethal, and all subunits of Ndc80 complex localize to centromeres/kinetochores throughout the cell cycle (Shin et al., 2018). A weak allele of MUN1 displays stunted growth, aneuploidy, and chromosome segregation defects and interaction of MUN1 with other Ndc80 complex components was confirmed through yeast two-hybrid assays and co-immunoprecipitation, establishing MUN1 as a functional homologue of Spc24 (Shin et al., 2018). Another study examined AtNuf2 role in partially complemented *nuf2* mutant seedlings, revealing mitotic defects like aberrant spindle microtubules, chromosome bridges, and lagging chromosomes (Li et al., 2021). In maize, a homologue of Ndc80 is consistently present on kinetochores during the cell cycle and located outside the inner kinetochore protein CENP-C during meiosis (Du and Dawe, 2007).

In animal cells, CENP-E, also known as kinesin 7, is a motor protein that plays an important role in chromosome congression and SAC activation (Maiato et al., 2017). A study in *P. patens* identifying kinesin7-III as a potential CENP-E due to its co-localization with Mis12 (Miki et al., 2014), and another in barley showing antibodies against Cpel1 and Cpel2 label centromeres on mitotic chromosomes (Hoopen et al., 2002). This knowledge gap was bridged by a recent study in Arabidopsis which identified kinetochore-associated kinesin 1 (KAK1) from the kinesin 7 family as a downstream target of BUB3.3 driving chromosome congression (Tang et al., 2024).

The SKA complex is another microtubule-binding kinetochore subunit conserved in many eukaryotes. In the moss *P*. *patens*, SKA1 and SKA2 proteins localize around the nucleus during prophase and are recruited to kinetochores after nuclear envelope breakdown. Knockdown of SKA1 leads to various chromosome missegregation defects and mitotic delay, indicating its crucial role in moss kinetochores (Kozgunova et al., 2019). While the SKA complex is also present in angiosperms, no functional analyses have been conducted to date.

### 1.3 Regulatory proteins: spindle assembly checkpoint complex and chromosome passenger complex

The spindle assembly checkpoint (SAC), composed of BUB and MAD proteins, ensures that chromosomes are properly attached to the spindle microtubules. In fungi and animals, at the start of cell division, MAD1 and MAD2 are recruited to kinetochores, followed by BUBR1/MAD3, which associates with kinetochores through interaction with the WD40 repeat protein BUB3. Together, BUB3, BUBR1/MAD3, and MAD2 form the mitotic checkpoint complex (MCC) with CDC20, the activator of the APC/C (Lara-Gonzalez et al., 2021).

Although the core framework of SAC components is also found in plants (Caillaud et al., 2009; Komaki and Schnittger, 2016), the BUB1/MAD3 family has undergone several duplication events, evolving in a different way from Bub1 and BubR1/Mad3 in yeast and animal. In recognition of their differences, new gene nomenclature has been established: BUB1/MAD3 FAMILY 1 (BMF1) for BUB1, BMF2 for MAD3.1, and BMF3 for MAD3.2 (Komaki and Schnittger, 2017). Surprisingly, the SAC significance in plant development under normal conditions appears to be minor. This is supported by the fact that homozygous DNA insertion mutants of all putative core SAC genes could be isolated in Arabidopsis (Ding et al., 2012; Komaki and Schnittger, 2017; Zhang et al., 2018). Although the mutants are more sensitive to microtubule-depolymerizing drugs, they grow similarly to wild-type plants under normal conditions (Komaki and Schnittger, 2017). SAC proteins also exhibit localization patterns distinct from mammalian and yeast cells; for example, BMF3 and MAD2 show typical accumulation at kinetochores post-nuclear envelope breakdown, while BMF1 and MPS1 localize to centromeric regions throughout the cell cycle. BUB3 proteins display unique plant-specific localization: notably, BUB3; 1 and BUB3; 2 are observed in the phragmoplast midzone during cytokinesis (Komaki and Schnittger, 2017). Mutations in BUB3; 1 were originally reported to cause embryonic lethality (Lermontova et al., 2008); however a later study could isolate both *bub3;1* mutant and *bub3;1/bub3;2* double mutant (Zhang et al., 2018). BUB3; 1 and BUB3; 2 were shown to interact with MAP65-3 and play a role in regulating phragmoplast microtubule dynamics by enhancing the binding of MAP65-3 to microtubules (Zhang et al., 2018). In Arabidopsis, BUB3; 3 is detected at kinetochores throughout mitosis, and *bub3;3* T-DNA insertion mutants often exhibit misaligned chromosomes and possess a non-functional SAC (Deng et al., 2024b). Another study discovered that chromosome misalignment in BUB3.3-depleted plants can be rescued by artificial tethering of KAK1 (kinesin-7) to kinetochores, suggesting KAK1 as a downstream target (Tang et al., 2024). Interestingly, the localization of SAC proteins, including MPS1, MAD1, BMF2, and BMF3, was found to be independent of BUB3; 3 recruitment to the kinetochore and *vice versa*. However, the interaction between BUB3; 3 and BMF3 enables the downstream recruitment of CDC20 to kinetochores (Deng et al., 2024b).

Another group of regulatory proteins which plays an important role in correcting kinetochore-microtubule attachments is the Chromosome Passenger Complex (CPC). The CPC resides mostly on inner centromere and consists of four proteins: the kinase Aurora B, INCENP, Borealin, and Survivin. In animal cells, Aurora B destabilizes incorrect kinetochore-microtubule attachments by selectively phosphorylating Ndc80 (Haase et al., 2017; Trivedi and Stukenberg, 2020). Plants also possess CPC, although with an altered composition. The homologue of Aurora B in plants is encoded by the AURORA 3 (AUR3) gene, which localizes to the centromeres and kinetochores (Demidov et al., 2005). A plant homologue of INCENP was identified through the study of the *wyrd* (*wyr*) mutant with abnormal ovule development (Kirioukhova et al., 2011). Further analysis revealed that both INCENP and homologue of Borealin, BOREALIN RELATED (BORR), localize to the centromere and kinetochore, similar to AUR3. Frameshift mutations in BORR are lethal, and knockdown of BORR compromises chromosome segregation and development (Komaki et al., 2020). Recently, two Survivin-like genes, BOREALIN RELATED INTERACTOR 1 and 2 (BORI1 and BORI2), were also identified in Arabidopsis. Loss of BORIs’ function is lethal, and reduced expression of BORIs leads to severe developmental defects. Similar to Survivin, BORIs are essential for targeting the CPC to chromatin and bind to phosphorylated histone H3 through their FHA domain (Komaki et al., 2022). Although CPC components have been identified in plants, their role in correcting kinetochore-microtubule attachments is still unclear, indicating a promising area for future research.

### 1.4 Non-canonical kinetochores in the *Cuscuta* parasitic plants

The plant kingdom includes both monocentric species with a single centromere per chromosome and holocentric species with centromere activity along the chromosome length (Chandra et al., 2024; Naish and Henderson, 2024). The genus *Cuscuta*, consisting of around 200 species of parasitic plants, includes both monocentric and holocentric species. This diversity provides a unique opportunity to investigate the changes associated with the transition from monocentric to holocentric in a closely related species. In the holocentric species *Cuscuta europaea*, two variants of CENH3 are located in one to three discrete regions per chromosome, while the rest of the chromatin lack CENH3 signals. Despite this distribution, spindle microtubules attach uniformly along the entire length of the chromosomes, including the CENH3-free areas. This raises the question of whether CENH3 has lost its function or operates alongside an alternative CENH3-free mechanism for kinetochore positioning (Oliveira et al., 2020). The transition to holocentric chromosomes is accompanied by drastic changes in kinetochore composition, most notably the loss of KNL2 and several SAC genes, while CENP-C, KNL1, and ZWINT-1 homologues are truncated. Furthermore, in *Cuscuta epithymum*, no CENH3 signal is detected on the chromosomes; consequently, the centromeric localization of kinetochore proteins CENP-C, KNL1, Mis12, and Ndc80 is disrupted (Neumann et al., 2023). This suggests that some holocentric *Cuscuta* species do not form a conventional kinetochore and have either evolved unique kinetochore genes, reminiscent of kinetoplastids (Akiyoshi, 2016), or developed alternative kinetochore assembly mechanisms, like *Lepidoptera* species utilize divergent CENP-T, instead of CENH3-CENP-C, for kinetochore assembly (Cortes-Silva et al., 2020).

## 2 Discussion

Although the centromere/kinetochore tandem performs a highly conserved function in eukaryotes, there is considerable variability in centromeric repeats and kinetochore complex composition across species (Roach et al., 2012). For instance, the inner kinetochore in plants consists of only a few proteins compared to the 16 subunits of the human CCAN. CENP-C appears to be the most functionally conserved inner kinetochore protein characterized across different plant species (Ogura et al., 2004; Nagaki et al., 2009; Du et al., 2010). While it is possible that plants have lost many inner kinetochore components, like *D. melanogaster* or *C. elegans* (Drinnenberg et al., 2016), another possibility is that plants possess additional, yet-to-be-identified proteins that contribute to the inner kinetochore structure. A promising avenue for future studies would be to use co-immunoprecipitation or proximity labelling techniques to investigate the full composition of the inner kinetochore in plants.

Recent studies have shown that differences in kinetochore architecture and function exist even within the plant kingdom, such as the unique evolution of KNL1 and its interactions with spindle checkpoint proteins, as well as the distinctive kinetochores in *Cuscuta* species. In angiosperms, most outer kinetochore proteins and several SAC proteins are constitutively observed on centromeres throughout the cell cycle. In contrast, in the bryophyte *P. patens*, only CENH3 and CENP-C remain on the centromere for most of the cell cycle. The KNL1 and Mis12 complexes appear in prophase, while the Ndc80 and SKA complexes are recruited only after nuclear envelope breakdown, suggesting a time-dependent kinetochore assembly. However, it remains unclear whether this localization pattern is specific to *P. patens* or reflects a broader trend among non-vascular plants.

Many aspects of microtubule binding by plant kinetochores also remain uncertain. While key microtubule-binding proteins such as CENP-E (KAK1, kinesin-7), the Ndc80 complex, and the SKA complex appear to be conserved, functional analyses of their interaction with microtubules remain limited. Notably, a recent study found that Arabidopsis Spc25 has a higher affinity for microtubule binding compared to Nuf2 (Li et al., 2021), despite Nuf2 and Ndc80 forming a microtubule-binding module in animal kinetochores. Adding to the complexity, most SAC proteins, which regulate mitotic delay until proper microtubule-kinetochore attachments are established, are dispensable for plant development under normal conditions—a sharp contrast to their essential roles in animal cells. Could this dispensability of SAC be explained by an exceptionally robust microtubule binding by plant kinetochores?

While significant progress has been made in recent years, many questions remain regarding the complexities of kinetochore architecture and function in plants. The creation of hybrids and synthetic chromosomes, both closely linked to centromere-kinetochore function, are rapidly advancing fields that offer strong motivation to continue studying plant kinetochores.

## References

[B1] Akiyoshi B. (2016). The unconventional kinetoplastid kinetochore: from discovery toward functional understanding. Biochem. Soc. Trans. 44, 1201–1217. 10.1042/BST20160112 27911702 PMC5095916

[B2] Allipra S. Anirudhan K. Shivanandan S. Raghunathan A. Maruthachalam R. (2022). The kinetochore protein NNF1 has a moonlighting role in the vegetative development of *Arabidopsis thaliana* . Plant J. 109, 1064–1085. 10.1111/tpj.15614 34850467

[B3] Ariyoshi M. Fukagawa T. (2023). An updated view of the kinetochore architecture. Trends Genet. 39, 941–953. 10.1016/j.tig.2023.09.003 37775394

[B4] Batzenschlager M. Lermontova I. Schubert V. Fuchs J. Berr A. Koini M. A. (2015). *Arabidopsis* MZT1 homologs GIP1 and GIP2 are essential for centromere architecture. Proc. Natl. Acad. Sci. U.S.A. 112, 8656–8660. 10.1073/pnas.1506351112 26124146 PMC4507256

[B5] Breuer C. Stacey N. J. West C. E. Zhao Y. Chory J. Tsukaya H. (2007). BIN4, a novel component of the plant DNA topoisomerase VI complex, is required for endoreduplication in *Arabidopsis* . Plant Cell. 19, 3655–3668. 10.1105/tpc.107.054833 18055605 PMC2174874

[B6] Caillaud M.-C. Paganelli L. Lecomte P. Deslandes L. Quentin M. Pecrix Y. (2009). Spindle assembly checkpoint protein dynamics reveal conserved and unsuspected roles in plant cell division. PLoS One 4, e6757. 10.1371/journal.pone.0006757 19710914 PMC2728542

[B7] Chandra J. R. Kalidass M. Demidov D. Dabravolski S. A. Lermontova I. (2024). The role of centromeric repeats and transcripts in kinetochore assembly and function. Plant J. 118, 982–996. 10.1111/tpj.16445 37665331

[B8] Chen Q. Zhang M. Pan X. Yuan X. Zhou L. Yan L. (2021). Bub1 and CENP-U redundantly recruit Plk1 to stabilize kinetochore-microtubule attachments and ensure accurate chromosome segregation. Cell. Rep. 36, 109740. 10.1016/j.celrep.2021.109740 34551298

[B9] Cortes-Silva N. Ulmer J. Kiuchi T. Hsieh E. Cornilleau G. Ladid I. (2020). CenH3-Independent kinetochore assembly in Lepidoptera requires CCAN, including CENP-T. Curr. Biol. 30, 561–572. 10.1016/j.cub.2019.12.014 32032508

[B10] Dangel N. J. Knoll A. Puchta H. (2014). MHF 1 plays F anconi anaemia complementation group M protein (FANCM)‐dependent and FANCM ‐independent roles in DNA repair and homologous recombination in plants. Plant J. 78, 822–833. 10.1111/tpj.12507 24635147

[B11] Demidov D. Van Damme D. Geelen D. Blattner F. R. Houben A. (2005). Identification and dynamics of two classes of aurora-like kinases in Arabidopsis and other plants. Plant Cell. 17, 836–848. 10.1105/tpc.104.029710 15722465 PMC1069702

[B12] Deng X. He Y. Tang X. Liu X. Lee Y.-R. J. Liu B. (2024a). A coadapted KNL1 and spindle assembly checkpoint axis orchestrates precise mitosis in Arabidopsis. Proc. Natl. Acad. Sci. U.S.A. 121, e2316583121. 10.1073/pnas.2316583121 38170753 PMC10786300

[B13] Deng X. Peng F. L. Tang X. Lee Y.-R. J. Lin H.-H. Liu B. (2024b). The *Arabidopsis* BUB1/MAD3 family protein BMF3 requires BUB3.3 to recruit CDC20 to kinetochores in spindle assembly checkpoint signaling. Proc. Natl. Acad. Sci. U.S.A. 121, e2322677121. 10.1073/pnas.2322677121 38466841 PMC10963012

[B14] Ding D. Muthuswamy S. Meier I. (2012). Functional interaction between the Arabidopsis orthologs of spindle assembly checkpoint proteins MAD1 and MAD2 and the nucleoporin NUA. Plant Mol. Biol. 79, 203–216. 10.1007/s11103-012-9903-4 22457071

[B15] Drinnenberg I. A. Henikoff S. Malik H. S. (2016). Evolutionary turnover of kinetochore proteins: a ship of theseus? Trends Cell. Biol. 26, 498–510. 10.1016/j.tcb.2016.01.005 26877204 PMC4914419

[B16] Du Y. Dawe R. K. (2007). Maize NDC80 is a constitutive feature of the central kinetochore. Chromosome Res. 15, 767–775. 10.1007/s10577-007-1160-z 17643192

[B17] Du Y. Topp C. N. Dawe R. K. (2010). DNA binding of centromere protein C (CENPC) is stabilized by single-stranded RNA. PLoS Genet. 6, e1000835. 10.1371/journal.pgen.1000835 20140237 PMC2816676

[B18] Girard C. Crismani W. Froger N. Mazel J. Lemhemdi A. Horlow C. (2014). FANCM-associated proteins MHF1 and MHF2, but not the other Fanconi anemia factors, limit meiotic crossovers. Nucleic Acids Res. 42, 9087–9095. 10.1093/nar/gku614 25038251 PMC4132730

[B19] Haase J. Bonner M. K. Halas H. Kelly A. E. (2017). Distinct roles of the chromosomal passenger complex in the detection of and response to errors in kinetochore-microtubule attachment. Dev. Cell. 42, 640–654. 10.1016/j.devcel.2017.08.022 28950102 PMC6260983

[B20] Hoopen R. Schleker T. Manteuffel R. Schubert I. (2002). Transient CENP-E-like kinetochore proteins in plants. Chromosome Res. 10, 561–570. 10.1023/a:1020962618696 12498345

[B22] Kirik V. Schrader A. Uhrig J. F. Hulskamp M. (2007). *MIDGET* unravels functions of the *Arabidopsis* topoisomerase VI complex in DNA endoreduplication, chromatin condensation, and transcriptional silencing. Plant Cell. 19, 3100–3110. 10.1105/tpc.107.054361 17951446 PMC2174703

[B23] Kirioukhova O. Johnston A. J. Kleen D. Kägi C. Baskar R. Moore J. M. (2011). Female gametophytic cell specification and seed development require the function of the putative *Arabidopsis* INCENP ortholog *WYRD* . Development 138, 3409–3420. 10.1242/dev.060384 21752930

[B24] Komaki S. Schnittger A. (2016). The spindle checkpoint in plants — a green variation over a conserved theme? Curr. Opin. Plant Biol. 34, 84–91. 10.1016/j.pbi.2016.10.008 27816818

[B25] Komaki S. Schnittger A. (2017). The spindle assembly checkpoint in Arabidopsis is rapidly shut off during severe stress. Dev. Cell. 43, 172–185. 10.1016/j.devcel.2017.09.017 29065308

[B26] Komaki S. Takeuchi H. Hamamura Y. Heese M. Hashimoto T. Schnittger A. (2020). Functional analysis of the plant chromosomal passenger complex. Plant Physiol. 183, 1586–1599. 10.1104/pp.20.00344 32461300 PMC7401102

[B27] Komaki S. Tromer E. C. De Jaeger G. De Winne N. Heese M. Schnittger A. (2022). Molecular convergence by differential domain acquisition is a hallmark of chromosomal passenger complex evolution. Proc. Natl. Acad. Sci. U.S.A. 119, e2200108119. 10.1073/pnas.2200108119 36227914 PMC9680938

[B28] Kozgunova E. Nishina M. Goshima G. (2019). Kinetochore protein depletion underlies cytokinesis failure and somatic polyploidization in the moss. eLife 43652. 10.7554/eLife.43652 PMC6433463 30835203

[B29] Lara-Gonzalez P. Pines J. Desai A. (2021). Spindle assembly checkpoint activation and silencing at kinetochores. Seminars Cell. and Dev. Biol. 117, 86–98. 10.1016/j.semcdb.2021.06.009 PMC8406419 34210579

[B30] Lermontova I. Fuchs J. Schubert I. (2008). The Arabidopsis checkpoint protein Bub3.1 is essential for gametophyte development. Front. Biosci. 13, 5202–5211. 10.2741/3076 18508582

[B31] Li J. Wang Y. Zou W. Jian L. Fu Y. Zhao J. (2021). *AtNUF2* modulates spindle microtubule organization and chromosome segregation during mitosis. Plant J. 107, 801–816. 10.1111/tpj.15347 33993566

[B32] Li Y. Zhou Y. Wang B. Mu N. Miao Y. Tang D. (2023). FANCM interacts with the MHF1‐MHF2 complex to limit crossover frequency during rice meiosis. Plant J. 116, 717–727. 10.1111/tpj.16399 37632767

[B33] Maiato H. Gomes A. Sousa F. Barisic M. (2017). Mechanisms of chromosome congression during mitosis. Biology 6, 13. 10.3390/biology6010013 28218637 PMC5372006

[B34] Marimuthu M. P. A. Maruthachalam R. Bondada R. Kuppu S. Tan E. H. Britt A. (2021). Epigenetically mismatched parental centromeres trigger genome elimination in hybrids. Sci. Adv. 7, eabk1151. 10.1126/sciadv.abk1151 34797718 PMC8604413

[B35] McAinsh A. D. Kops G. J. P. L. (2023). Principles and dynamics of spindle assembly checkpoint signalling. Nat. Rev. Mol. Cell. Biol. 24, 543–559. 10.1038/s41580-023-00593-z 36964313

[B36] Miki T. Naito H. Nishina M. Goshima G. (2014). Endogenous localizome identifies 43 mitotic kinesins in a plant cell. Proc. Natl. Acad. Sci. U.S.A. 111, E1053–E1061. 10.1073/pnas.1311243111 24591632 PMC3964101

[B37] Musacchio A. Desai A. (2017). A molecular view of kinetochore assembly and function. Biology 6, 5. 10.3390/biology6010005 28125021 PMC5371998

[B38] Nagaki K. Kashihara K. Murata M. (2009). Characterization of the two centromeric proteins CENP-C and MIS12 in Nicotiana species. Chromosome Res. 17, 719–726. 10.1007/s10577-009-9064-8 19697146

[B39] Naish M. Henderson I. R. (2024). The structure, function, and evolution of plant centromeres. Genome Res. 34, 161–178. 10.1101/gr.278409.123 38485193 PMC10984392

[B40] Neumann P. Oliveira L. Jang T.-S. Novák P. Koblížková A. Schubert V. (2023). Disruption of the standard kinetochore in holocentric Cuscuta species. Proc. Natl. Acad. Sci. U.S.A. 120, e2300877120. 10.1073/pnas.2300877120 37192159 PMC10214151

[B41] Nguyen A. L. Fadel M. D. Cheeseman I. M. (2021). Differential requirements for the CENP-O complex reveal parallel PLK1 kinetochore recruitment pathways. MBoC 32, 712–721. 10.1091/mbc.E20-11-0751 33596090 PMC8108507

[B42] Ogura Y. Shibata F. Sato H. Murata M. (2004). Characterization of a CENP-C homolog in *Arabidopsis thaliana* . Genes. Genet. Syst. 79, 139–144. 10.1266/ggs.79.139 15329494

[B43] Oliveira L. Neumann P. Jang T.-S. Klemme S. Schubert V. Koblížková A. (2020). Mitotic spindle attachment to the holocentric chromosomes of Cuscuta europaea does not correlate with the distribution of CENH3 chromatin. Front. Plant Sci. 10, 1799. 10.3389/fpls.2019.01799 32038700 PMC6992598

[B44] Pesenti M. E. Raisch T. Conti D. Walstein K. Hoffmann I. Vogt D. (2022). Structure of the human inner kinetochore CCAN complex and its significance for human centromere organization. Mol. Cell. 82, 2113–2131.e8. 10.1016/j.molcel.2022.04.027 35525244 PMC9235857

[B45] Puchta H. Houben A. (2024). Plant chromosome engineering – past, present and future. New Phytol. 241, 541–552. 10.1111/nph.19414 37984056

[B46] Quiroz L. F. Gondalia N. Brychkova G. McKeown P. C. Spillane C. (2024). Haploid rhapsody: the molecular and cellular orchestra of *in vivo* haploid induction in plants. New Phytol. 241, 1936–1949. 10.1111/nph.19523 38180262

[B58] Roach K. C. Ross B. D. Malik H. S. (2012). “Rapid evolution of centromeres and centromeric/kinetochore proteins,” in Rapidly Evolving Genes and Genetic Systems. Editors Singh R. S. Xu J. Kulathinal R. J. (Oxford University Press), 83–93. 10.1093/acprof:oso/9780199642274.003.0009

[B47] Sato H. Shibata F. Murata M. (2005). Characterization of a Mis12 homologue in *Arabidopsis thaliana* . Chromosome Res. 13, 827–834. 10.1007/s10577-005-1016-3 16331414

[B48] Shin J. Jeong G. Park J. Kim H. Lee I. (2018). Mun (meristem unstructured), encoding a SPC24 homolog of NDC80 kinetochore complex, affects development through cell division in *Arabidopsis thaliana* . Plant J. 93, 977–991. 10.1111/tpj.13823 29356153

[B49] Singh P. Pesenti M. E. Maffini S. Carmignani S. Hedtfeld M. Petrovic A. (2021). BUB1 and CENP-U, primed by CDK1, are the main PLK1 kinetochore receptors in mitosis. Mol. Cell. 81, 67–87.e9. 10.1016/j.molcel.2020.10.040 33248027 PMC7837267

[B50] Singh T. R. Saro D. Ali A. M. Zheng X.-F. Du C. Killen M. W. (2010). MHF1-MHF2, a histone-fold-containing protein complex, participates in the fanconi anemia pathway via FANCM. Mol. Cell. 37, 879–886. 10.1016/j.molcel.2010.01.036 20347429 PMC2848122

[B51] Su H. Liu Y. Wang C. Liu Y. Feng C. Sun Y. (2021). Knl1 participates in spindle assembly checkpoint signaling in maize. Proc. Natl. Acad. Sci. U.S.A. 118, e2022357118. 10.1073/pnas.2022357118 33990465 PMC8157932

[B52] Tang X. He Y. Tang Y. Chen K. Lin H. Liu B. (2024). A kinetochore-associated kinesin-7 motor cooperates with BUB3.3 to regulate mitotic chromosome congression in *Arabidopsis thaliana* . Nat. Plants 10, 1724–1736. 10.1038/s41477-024-01824-7 39414927

[B53] Trivedi P. Stukenberg P. T. (2020). A condensed view of the chromosome passenger complex. Trends Cell. Biol. 30, 676–687. 10.1016/j.tcb.2020.06.005 32684321 PMC10714244

[B54] Tromer E. Snel B. Kops G. J. P. L. (2015). Widespread recurrent patterns of rapid repeat evolution in the kinetochore scaffold KNL1. Genome Biol. Evol. 7, 2383–2393. 10.1093/gbe/evv140 26254484 PMC4558858

[B55] Wang M. L. Lin X. J. Mo B. X. Kong W. W. (2024). Plant artificial chromosomes: construction and transformation. ACS Synth. Biol. 13, 15–24. 10.1021/acssynbio.3c00555 38163256

[B56] Yan K. Yang J. Zhang Z. McLaughlin S. H. Chang L. Fasci D. (2019). Structure of the inner kinetochore CCAN complex assembled onto a centromeric nucleosome. Nature 574, 278–282. 10.1038/s41586-019-1609-1 31578520 PMC6859074

[B57] Zhang H. Deng X. Sun B. Lee Van S. Kang Z. Lin H. (2018). Role of the BUB3 protein in phragmoplast microtubule reorganization during cytokinesis. Nat. Plants 4, 485–494. 10.1038/s41477-018-0192-z 29967519

